# Knowledge, attitudes, and practices concerning cockroach exposure among caregivers of children with asthma

**DOI:** 10.1186/s12889-021-11497-y

**Published:** 2021-07-29

**Authors:** Derek Werthmann, Felicia A. Rabito, Charlie Reed

**Affiliations:** Department of Epidemiology, Tulane University School of Public Health and Tropical Medicine, New Orleans, LA USA

**Keywords:** Cockroach, Knowledge, Attitudes, Practices, Asthma, Health beliefs model

## Abstract

**Background:**

Cockroach allergen is one of the most important asthma triggers for children. There is an extensive body of research on interventions to reduce exposure. However, adherence to these interventions is low. Insight into the knowledge, attitudes, and practices (KAP) associated with cockroach remediation is needed. We assessed KAP using the Health Beliefs Model (HBM) as a framework for predicting behavior. This study aimed to assess the socio-demographic and psycho-social characteristics associated with cockroach KAP and to quantify the relationship between KAP and number of cockroaches in the home.

**Methods:**

To identify factors associated with cockroach KAP a cross-sectional study was conducted using a structured questionnaire administered to caregivers of children with asthma in New Orleans, Louisiana. Positive scores in each metric of the KAP signified better cockroach knowledge, increased concern about cockroaches’ impact on health (attitudes), and participation in recommended cockroach remediation practices (practices). To evaluate cockroach KAP scores as a predictor of cockroach exposure in the home, a cohort study was conducted among a sub-sample of participants.

**Results:**

Fifty-six caregivers participated in the study. Participants had positive scores on all subscales of KAP with knowledge having a lower score compared to attitudes and practices. Cockroach knowledge was inversely correlated with age at asthma diagnosis (*ρ* = − 0.36, *p* = 0.01). Caregivers identifying as black/African American had higher cockroach knowledge scores compared to other races (Median: 6.0 v 3.0; *p* = 0.05). Caregivers other than mothers had higher cockroach attitude scores (Median 6.5 v 4.0; *p* = 0.003) and total KAP scores (Median 18.0 v 14.0; *p* = 0.05). Twenty-six participants completed the cohort study. Cockroach exposure was not significantly associated with higher cockroach knowledge, cockroach practice, or total KAP score. For attitude scores, participants in the highest quartile had significantly lower exposure (β: -1.96, 95% CI: − 3.50 - 0.42) compared to those in the lowest quartile (*p* = 0.01).

**Conclusions:**

Different socio-demographic and psycho-social factors were associated with the components of cockroach KAP. Greater concern about cockroaches (attitude) was significantly associated with reduced cockroach exposure. This highlights the importance of identifying the key elements of caregiver KAP to improve cockroach remediation among caregivers of children with asthma.

## Background

In the United States (U.S.) asthma is the most common childhood disease with an estimated prevalence of 6 million [1]. Asthma is also the leading cause of childhood morbidity [2] and a major contributor to school absenteeism with over 5.2 million lost school days annually due to asthma [3]. Families of low-income status are disproportionately affected. Compared to a prevalence of 8.3% in the general U.S. population, for those living below the federal poverty threshold the prevalence is 11.8% [4]. Clinical guidelines outline best practices for preventing asthma exacerbations and an important component is cockroach allergen avoidance. However, adherence to cockroach avoidance strategies is low (12–40%) even when resources are supplied [5]. The reasons for low adherence are unclear.

Exposure to high levels of allergens in the home is related to poor asthma outcomes. Of the indoor allergens known to be related to asthma, exposure to cockroach, which is ubiquitous in urban environments, is strongly associated with severe asthma outcomes in children of low-income status [6–8]. Cockroach exposure is associated with significantly higher rates of hospitalization [9] and children sensitized and highly exposed to cockroach have almost double the number of unscheduled medical visits, and significantly more days of wheezing (4.04 v 3.21) and missed school (7.65 v 6.12) [10]. Cockroach allergen has been detected in 85% of inner-city homes in the U.S. and an estimated 60–80% of inner-city children with asthma are sensitized to cockroach [11]. Asthma management is complex requiring a multifaceted approach of both medication and environmental interventions. The most important environmental intervention is controlling exposure to allergens in the home [12]. It is an effective asthma management strategy; shown to be as effective at improving asthma outcomes as asthma medication [13]. However, data from the National Health Interview Survey show that less than half (46%) of caregivers of children with asthma receive advice on environmental control [14]. Research also shows caregivers are not willing to adhere to the recommended interventions [15]. While there is a robust body of research examining factors related to medication adherence [16], research on the factors related to adherence to recommended environmental interventions, including cockroach remediation, is lacking. Given the effectiveness of cockroach remediation on improved asthma morbidity [17], this is an important knowledge gap.

Knowledge attitudes and practices (KAP) surveys are conducted to obtain health behavior information about what is known (knowledge), what is thought (attitudes), and what is done (practices) in specific populations. KAP surveys can identify knowledge gaps, cultural beliefs, behavioral patterns, and barriers. This information can be used to deepen the understanding of behaviors and plan the implementation of interventions [18]. Despite abundant data on the importance of cockroach allergen avoidance for children with asthma, the KAP regarding cockroach avoidance strategies and the impact cockroach KAP has on actual exposure in the home is unknown.

The Health Belief Model (HBM) was developed in the 1950s by the U.S. Public Health Services to determine why people do not adopt preventative health measures [19]. It theorizes that health behavior is determined by personal beliefs, perceptions about the disease, and the availability of strategies to prevent its occurrence. The HBM’s effectiveness in predicting behavior is well-documented [20, 21]. The core of the HBM is an individual’s perception of disease which is influenced by sociodemographic and psychosocial components. The HBM has six core constructs: perceived severity, perceived susceptibility, cues to action, perceived benefits, perceived barriers, and self-efficacy. Perceived severity of the disease and perceived susceptibility both contribute to an individual’s perception of threat. Threat perception, along with perceived benefits, perceived barriers and self-efficacy determine the likelihood of a behavior being carried out [22]. Cues to action are independent triggers, either internal or external, that prompt an individual to action. We used the HBM as a framework to assess caregiver’s qualities (sociodemographic and psychosocial) associated with cockroach KAP in order to explore the reasons for low adherence to cockroach avoidance strategies. We then assessed the impact of KAP regarding cockroaches on the level of cockroach exposure in the home.

The objective of this study was to describe the socio-demographic and psychosocial factors associated with cockroach KAP. A secondary objective was to assess the association between caregiver’s cockroach KAP and actual exposure to cockroach in their home. The study hypothesis is that greater knowledge about cockroach (knowledge), higher concern about cockroach exposure (attitudes), and actions that promote cockroach remediation (practices) will result in reduced exposure to cockroaches in the home.

## Methods

**Fig. 1:**
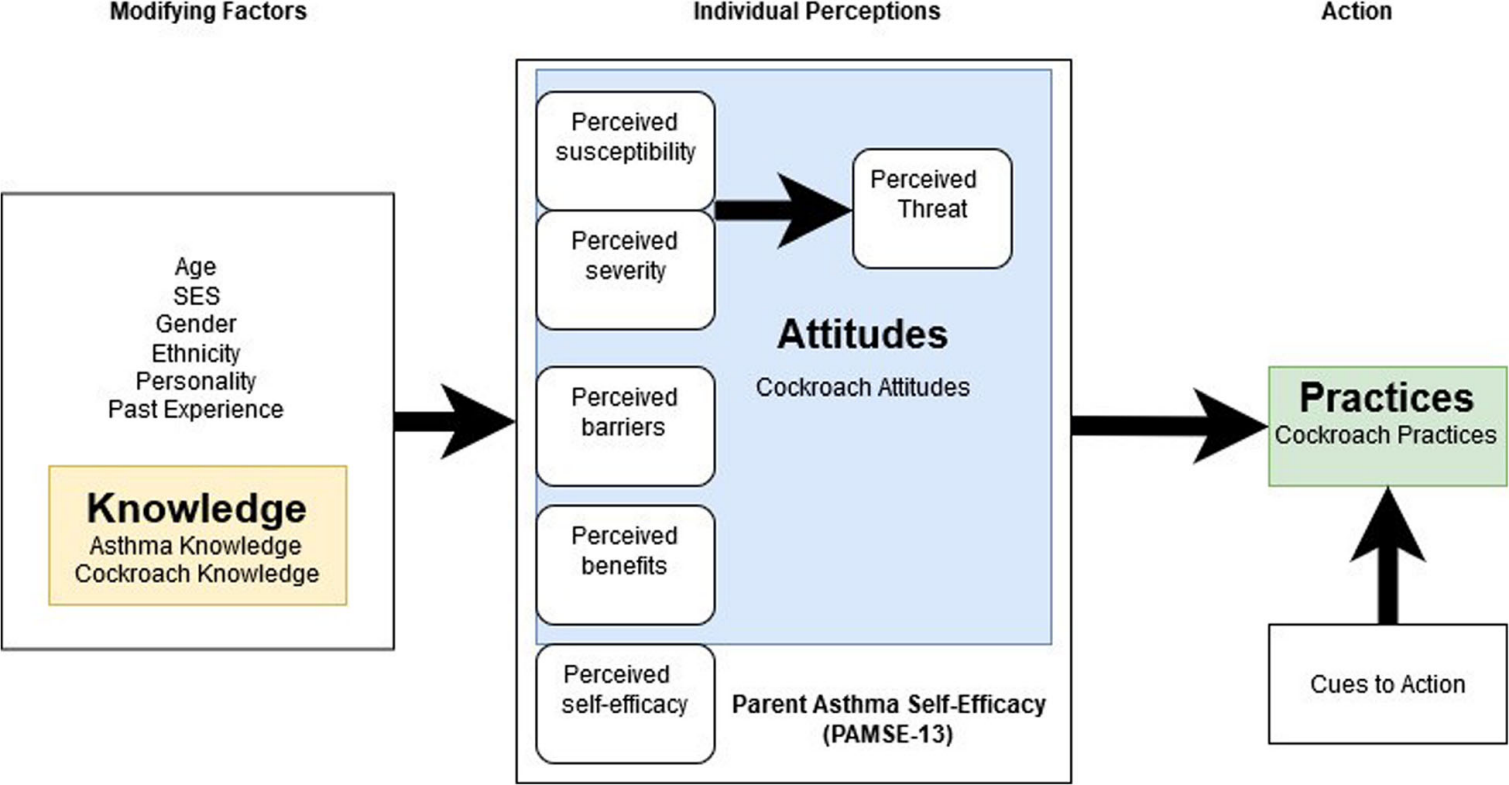
Health Beliefs Model as an explanatory framework for cockroach remediation

Using the HBM framework, (Fig. 1) we assessed the socio-demographic and psychosocial factors associated with cockroach KAP. Key socio-demographic factors include: race/ethnicity, education, marital status and housing factors (home ownership status, type of dwelling). Key psychosocial factors include stress, asthma knowledge, self-efficacy, social support, and medication beliefs.

### Study design

A cross-sectional study was performed to examine KAP of caregivers of children with asthma regarding the role of cockroach remediation on their child’s asthma and to examine the correlation between cockroach KAP and specific socio-demographic and psycho-social factors. A cohort study was conducted among a sub-sample of participants to determine whether cockroach KAP scores are associated with cockroach exposure in the home. The cohort study consisted of administering the KAP survey followed by a home visit to measure cockroach counts. The study received approval from the Tulane University Biomedical Institutional Review Board and informed consent was obtained prior to data collection.

### Study participants

Participants were recruited from community health fairs and the Children’s Hospital of New Orleans Pediatric Pulmonary Clinic between August 2018 and April 2019. Eligibility requirements included being the caregiver of a child (< 18 years) with doctor-diagnosed asthma.

### Data collection

#### Knowledge, attitude and practices

Knowledge, Attitude and Practices concerning cockroaches and their effect on asthma was assessed via an interviewer administered questionnaire adapted from a previous cockroach intervention study conducted in Toronto, Canada [23]. Study personnel trained in survey data collection administered the questionnaire. The KAP survey consists of three subscales: knowledge, attitudes and practices (Table 1). Knowledge parameters included health risks associated with exposure to cockroaches, factors contributing to cockroach infestation, and health risks associated with use of insecticides. The knowledge subscale consists of 20 statements with responses of “agree, disagree, and not sure”. Correct responses were scored as 1 point and incorrect responses or a response of “not sure” were scored as negative 1, resulting in a scale ranging from − 20 to 20. Positive scores represent increasing knowledge while negative scores signify increasing inaccurate knowledge. The attitudes subscale of the KAP survey covered perceived effectiveness of cockroach control, health concerns about spraying insecticides to control cockroaches, and overall concern about cockroach exposure in the home. The attitudes scale consists of 10 statements with 4-item responses of “strongly agree, agree, disagree, or strongly disagree”. Each item was scored as either − 1, − 0.5, 0.5, or 1.0. The resulting scale ranges from − 10 to 10 with increasing values being associated with greater acceptance of cockroach mitigation/remediation and greater concern about health risks associated with cockroaches. Finally, the KAP survey assessed caregivers’ practices regarding the management and prevention of cockroach infestation. The practices scale consists of 10 items with 3-item responses of “often, sometimes, or rarely”, these items were scored as − 1, 0, or 1. The resulting scale ranges from − 10 to 10 with positive values signifying practices for avoiding cockroaches and negative values indicating more practices that promoted cockroach infestation. The overall KAP score is the sum of the 3 sub-scales, ranging from − 40 to 40.

**Table 1:** Cockroach KAP questions and relationship to Health Beliefs Model

Knowledge	Health Beliefs Model Construct
**1. Health risks of pesticides**	
a. Exposure to some pesticides may interfere with the nervous system.	Knowledge
b. Smelling an odor does not necessarily mean over-exposure to pesticides.	Knowledge
c. Long-term exposure to pesticides, at low levels, is not safe.	Knowledge
d. Boric acid can be hazardous.	Knowledge
**2.** The following are **health risks and habits of cockroaches:**	
a. Contact with cockroaches does not hurt skin.	Knowledge
b. Cockroaches do not give off harmful vapors.	Knowledge
c. Cockroaches do not worsen Tuberculosis conditions.	Knowledge
d. About 10% of people are allergic to cockroaches.	Knowledge
e. Persons with asthma are much more likely to have allergic reactions to cockroaches than non-asthmatics.	Knowledge
f. Cockroaches prefer dark, tight spaces.	Knowledge
**3.** These **factors contribute to cockroach infestation:**	
a. Leaving grease on the stove overnight.	Knowledge
b. Leaving dirty dishes out overnight.	Knowledge
c. Keeping windows open.	Knowledge
d. Leaving food debris on the floor overnight.	Knowledge
e. Storing food in open packages in the cupboard.	Knowledge
f. Having a leaky tap.	Knowledge
g. Having a toilet that runs all the time.	Knowledge
h. Storing open bags of pet food.	Knowledge
i. Not having cracks or holes sealed around plumbing.	Knowledge
**4. Familiarity with cockroach bait method**	
a. Cockroach bait is one type of pest control method	Knowledge
**5. Effectiveness/desirability of alternatives**	
a. Cockroach baiting can be just as effective as spraying in controlling roaches.	Attitudes: Perceived Barriers
b. Cockroach baiting can be just as convenient as spraying in controlling roaches.	Attitudes: Perceived Barriers
c. I support safer methods other than spraying, even if they are slower-acting.	Attitudes: Perceived Barriers
d. I expect the safest method of pest control to be used, even if it is twice as expensive.	Attitudes: Perceived Barriers
**6. Concern about spraying**	
a. I have concerns about spraying in the home.	Attitudes: Perceived Benefits
b. Spraying is not the best way to eliminate cockroaches.	Attitudes: Perceived Benefits
**7. Concern about Cockroaches**	
a. I have concerns about health risks from cockroach infestation.	Attitudes: Perceived Susceptibility
b. I have concerns about seeing a cockroach in my home.	Attitudes: Perceived Severity
c. I have concerns about cockroaches in my home, even if not visible.	Attitudes: Perceived Severity
d. Cockroaches may be a problem, even in homes where people are tidy.	Attitudes: Perceived Susceptibility
**8. Recognition/management of cockroach problems**	
a. I would not just put up with cockroach problems; I would do something.	Practice
b. I would not use my own pest control product such as a spray or bait.	Practice
**9. Prevention of cockroach infestation**	
a. I clean crumbs soon after eating.	Practice
b. I clean dishes soon after eating.	Practice
c. I sweep the kitchen floor before bedtime.	Practice
d. I store food containers in the cupboard.	Practice
e. I clean grease off the stove before bedtime.	Practice
f. I rinse food off dishes if left unwashed overnight.	Practice
g. I thoroughly clean the kitchen/bathroom weekly.	Practice
h. I watch for cockroaches which might be brought home with groceries.	Practice

#### Cockroach exposure

Cockroach exposure was measured in a subsample of participants to assess the association between KAP score and the presence of cockroaches in the home. Cockroach exposure was defined as the number of cockroaches trapped (counts) in the home over a three-day period. Cockroach count data were obtained during a home visit that occurred within seven days of the participant’s completion of the KAP survey. Cockroach counts were obtained by study staff following protocols developed by researchers at North Carolina State University [24]. Briefly, an initial inspection of the home was performed, and study staff drew a layout map of the kitchen, living room, and the bedroom of the child with asthma. Next, a visual assessment of the home was conducted for any signs of cockroaches. The location and approximate number were recorded. Study personnel placed six pheromone sticky traps (Victor Roach Pheromone Traps, Woodstream, Lititz PA) in each room. The position of the traps was documented on the room diagram. Study personnel returned three days later to retrieve the traps. Traps were brought to the study laboratory where cockroaches were speciated and enumerated by trained study personnel. Data were recorded on a trap collection form.

#### Socio-demographic data

To assess the factors influencing cockroach KAP, socio-demographic and household data were collected via survey questionnaire using a structured instrument administered by study personnel. Data collected included: race/ethnicity of the caregiver, caregiver education level and marital status, type of residence, and age of the child at asthma diagnosis. The questionnaires were administered in person at the time of recruitment.

#### Psycho-social measures

Psychosocial factors associated with childhood asthma management were assessed using validated survey instruments [25–28] administered by study personnel either at the time of enrollment or at the home visit. To assess caregivers’ beliefs about asthma medication, an important component of asthma management, the Beliefs about Medicine Questionnaire (BMQ) was administered. The BMQ assesses parents’ beliefs and worries about children taking medication for their disease. It is an 18-item questionnaire with a four-factor structure (Specific-Necessity, Specific-Concerns, General-Harm, and General-Overuse). All items have a 5 point Likert answer option. Higher scores indicate stronger beliefs about the corresponding subscale (i.e. positive beliefs concerning medication use and negative beliefs about medication for the concerns, harm, and overuse scales). Social support is an important factor when carrying out health related behaviors. Therefore, the Medical Outcomes Study Social Support Survey Scale (MOS-SSS) was administered to each caregiver. The MOS-SSS assesses the extent to which a person has the support of others to face stressful situations. It consists of 6 items answered by means of a five-point Likert scale with scores ranging from 6 to 30. Higher scores indicating greater social support. Self-efficacy is one of the six key constructs of the HBM. To assess this construct the Parent Asthma Management Self-efficacy survey (PAMSE-13) was administered. The PAMSE-13 is a 13-item scale, consisting of two sub-scales, asthma management and asthma prevention, with 5-point Likert responses ranging from 13 to 65. Higher scores indicating greater self-efficacy for each sub-scale. Caregiver stress has a negative impact on asthma outcomes and the ability to perform asthma management behaviors [29]. Caregiver stress was measured via Cohen’s Perceived Stress Scale (PSS) short-form, a 4-item scale scored with 4-point Likert scale. Scores range from 0 to 16 with higher scores indicating greater perceived stress.

#### Asthma knowledge

Knowledge is a key construct of the HBM, and a caregiver’s knowledge of asthma is an important consideration when assessing cockroach KAP. Asthma knowledge was assessed using a questionnaire developed by Martinez et al. [30] The tool has been found to be reliable for quantifying the baseline level of asthma knowledge of parents of children with asthma. It consists of 17 questions and is scored on a 5-point Likert scale. Scores range from 17 to 85 with higher scores signifying greater asthma knowledge.

### Statistical analysis

Medians and inter quartile ranges are presented for continuous variables and the number and proportion of participants are presented for categorical variables. Spearman’s correlation coefficients are presented to assess the relationship between continuous variables (PSS, child age at asthma diagnosis, BMQ sub scales, PAMSE-13 sub scales, MOS-SSS scale and asthma knowledge score) and cockroach KAP. Differences in KAP score by categorical variables, race (black/AA v other), caregiver relationship to child (mother v other), caregiver education level (greater than high school, high school, or less than high school), home ownership (rent v own), seen roaches in the previous year (Yes v No), and type of dwelling (detached house, attached house, apartment) were analyzed using the Kruskal-Wallis test. Cockroach KAP scores were categorized into quartiles and associations with natural log transformed cockroach counts were assessed using linear regression. For all analyses, two sided tests were assumed and *p*-values < 0.05 were considered significant.

## Results

Fifty-six caregivers were recruited into the study (Table 2). The majority identified as Black/African American (88%) and were mothers of the child with asthma (72%). Of the participants that agreed to a home visit (*N* = 32), most reported renting their home (75%) and almost all reported seeing cockroaches in their home in the previous year (90%). Participants had positive scores on all subscales of the cockroach KAP survey with knowledge having a relatively lower score than attitudes and practices. For the BMQ the highest score was in the necessity scale (19 out of 25) with the harm scale having the lowest relative score (12 out of 20). The correlation between psycho-social and socio-demographic variables and KAP score are presented in Table 3. Cockroach knowledge scores were significantly inversely correlated with age at asthma diagnosis. Cockroach attitude scores were significantly correlated with the BMQ necessity scale, and cockroach practice scores had a significantly moderate correlation with PAMSE-13 attack management sub-scale. The total KAP scale was also significantly correlated with PAMSE-13 attack management scale. Total KAP score was not significantly associated with the concern, overuse, or harm BMQ subscales. The BMQ concern, overuse, and harm scales all had significant inverse correlations with asthma knowledge. For categorical variables (Table 4) caregivers identifying as Black/African American had higher cockroach knowledge scores compared to other races (Median: 6.0 v 3.0; *p* = 0.05) and caregivers other than mothers had higher cockroach attitudes (Median 6.5 v 4.0; *p* = 0.003) and total KAP scores (Median 18.0 v 14.0; *p* = 0.05). Twenty-six participants completed the home visit (Table 5). The number of cockroaches in the home was not associated with greater cockroach knowledge, cockroach practice or total KAP score. The highest quartile consistently had fewer cockroaches compared to the lowest quartile although results did not reach statistical significance. For cockroach attitude scores, participants in the highest quartile had significantly fewer cockroaches trapped (β: -1.96, 95% CI: − 3.50, − 0.42) compared to those with scores in the lowest quartile (*p* = 0.01).

**Table 2:** Characteristics of participants (*N* = 56)

	n	Median (IQR)
Age of Child at Asthma Diagnosis	51	3 (1–6)
Roach Number	26	21.5 (8–80)
Perceived Stress Scale	26	7 (4–10)
Medicine Beliefs Questionnaire	55	
Necessity Scale		19 (14–22)
Concerns Scale		14 (10–19)
Overuse Scale		14 (10–16)
Harm Scale		12 (10–13)
Asthma Knowledge	55	54 (49–57)
Cockroach KAP	56	
Knowledge Scale		6 (2–8)
Attitude Scale		4.75 (2.25–6.00)
Practice Scale		6.5 (4.0–8.0)
Cockroach KAP Total		15.25 (10–19.25)
Asthma Self-Efficacy	26	
Management		32 (30–34)
Prevention		28 (26–30)
Social Support	26	28.5 (25.0–30.0)
	n	n (%)
Race/Ethnicity	56	
Hispanic all Races		3 (5.35)
White		5 (8.93)
Black/African American		49 (87.5)
Other		2 (3.57)
Relationship to Child	54	
Mother		39 (72.2)
Father		4 (7.4)
Grandmother		10 (18.5)
Aunt		1 (1.85)
Education	56	
Less than High School		7 (12.5)
High School/GED		21 (37.5)
Greater than High School		28 (50.0)
Marital Status	56	
Married		9 (16.1)
Divorced		9 (16.1)
Single		37 (66.1)
Widowed		1 (1.79)
Home Ownership		
Rent Home	32	24 (75.0)
Seen Roaches Past Year	30	27 (90.0)
Type of Dwelling	31	
Detached House		12 (38.7)
Duplex/Triplex		11 (35.5)
Row House		2 (6.5)
Low Rise Apartment		6 (19.4)

**Table 3:** Spearman Correlation Coefficients of CR-KAP, BMQ, PAMSE-13, asthma knowledge, MOS-SSS, PSS, and age at asthma diagnosis

	CR Knowledge												
**CR Attitudes**	0.13	**CR Attitude**s											
**CR Practices**	0.03	0.10	**CR Practice**s										
**CR KAP**	0.69^***^	0.53^***^	0.50^***^	**CR KAP**									
**PSS**	0.27	0.09	−0.27	0.09	**PSS**								
**Age Asthma Diagnosis**	−0.36^***^	−0.01	0.02	−0.22	0.18	**Age Asthma Diagnosis**							
**Necessity** ^a^ **Scale**	0.08	0.28^***^	0.12	0.14	0.02	−0.24^*^	**Necessity Scale**						
**Concern** ^a^ **Scale**	0.22	0.02	0.13	0.10	0.30	0.04	0.41***	**Concern Scale**					
**Overuse** ^a^ **Scale**	−0.04	0.01	− 0.08	− 0.01	0.24	0.20	− 0.21	0.20	**Overuse Scale**				
**Harm Scale** ^a^	−0.02	−0.16	0.06	−0.03	0.55***	0.13	−0.26*	0.28**	0.65***	**Harm Scale**			
**Attack** ^b^ **Management**	0.30	−0.10	0.59^***^	0.43***	0.14	−0.09	−0.22	0.05	0.01	0.22	**Attack Management**		
**Attack** ^b^ **Prevention**	−0.01	0.07	0.37^*^	0.32	−0.32	−0.06	− 0.13	−0.08	− 0.03	−0.05	**0.43**	**Attack Prevention**	
**MOS-SSS**^c^	−0.01	0.07	0.22	0.24	−0.40**	−0.17	0.34*	0.19	−0.18	−0.28	0.33*	0.43^**^	**MOS-SSS**
**Asthma Knowledge**	0.08	−0.14	−0.01	0.04	−0.04	0.03	−0.15	− 0.48***	−0.37***	− 0.44***	0.18	0.15	−0.21

* *p* < 0.1; **p < 0.05: ***p < 0.01

CR: Cockroach; KAP: Knowledge, Attitudes, Practices PSS: Perceived Stress Scale; MOS-SSS: Social Support Survey

a Beliefs about Medicine Questionnaire (BMQ)

b Parental Asthma Management Self-Efficacy (PAMSE-13)

c Medical Outcome Study Social Support Scale

**Table 4:** Bivariate Association of CR-KAP and Sociodemographic factors

	CR Knowledge		CR Attitudes		CR Practices		CR KAP Total	
	Median (95% CI)	p	Median (95% CI)	p	Median (95% CI)	p	Median (95% CI)	p
Race/Ethnicity		0.05		0.11		0.14		0.84
Black/African American	6 (4–6)		4 (3.5–5)		6 (6–8)		15 (12.5–17)	
Other	3 (−4–4)		5.5 (4.5–9)		8 (2–10)		16 (9.5–21)	
Relationship to child		0.62		0.003		0.28		0.05
Mother	6 (2–6)		4 (3–5)		6 (5–8)		14 (11–16)	
Other	4 (4–8)		6.5 (5–8)		8 (6–8)		18 (15–20.5)	
Education		0.81		0.16		0.09		0.54
Less than HS	4 (−4–8)		4 (2–6)		8 (6–10)		16 (11–21.5)	
HS	4 (2–6)		3 (2–6)		6 (4–8)		14.5 (9.5–16.5)	
Greater than HS	6 (4–6)		5 (4–6)		6 (4–8)		16.5 (10–19.5)	
Partnered		0.22		0.52		0.32		0.5
Yes	8 (0–8)		5 (1.5–7.5)		6 (4–8)		17 (9.5–20.5)	
No	4 (2–6)		4.5 (3.5–5)		7 (6–8)		15 (12–16.5)	
Home Ownership		0.64		0.69		0.64		0.79
Own Home	5 (2–10)		4.25 (2–8.5)		7 (3–10)		15.25 (11–26.5)	
Rent Home	6 (2–6)		4 (3–5)		7.5 (6–8)		15.5 (12–18)	
Seen Roaches Past Year		0.81		0.43		0.73		0.78
Yes	6 (2–6)		4 (3–5)		7 (6–8)		15 (12–18)	
No	6 (2–8)		3.5 (3–3.5)		7 (4–10)		13.5 (12–21.5)	
Type of Dwelling		0.83		0.79		0.19		0.19
Detached House	4 (2–8)		4.25 (2–6.5)		7 (4–8)		16 (10–20.5)	
Attached House	6 (−4–8)		4 (2–5)		6 (3–8)		15 (7–17)	
Apartment	6 (2–8)		4.25 (1.5–8)		8 (3–10)		18.25 (12–22)

**Table 5:** Association between natural log roach number and CR-KAP Scales

	β (95% CI)	p
Cockroach Knowledge		0.82
Q1	Ref	
Q2	0.10 (− 2.67, 2.86)	
Q3	0.59 (− 2.12, 3.3)	
Q4	−0.26 (− 3.12, 2.59)	
Cockroach Attitudes		**0.01**
Q1	Ref	
Q2	−1.69 (−3.49, 0.12)	
Q3	0.49 (−1.00, 1.98)	
Q4	−1.96 (−3.50, −0.42)	
Cockroach Practices		0.43
Q1	Ref	
Q2	−1.73 (−3.99, 0.54)	
Q3	−1.48 (−3.40, 0.43)	
Q4	−1.65 (−4.23, 0.93)	
CR-KAP Total		0.57
Q1	Ref	
Q2	−0.30 (−2.72, 2.12)	
Q3	0.23 (−1.83, 2.29)	
Q4	−0.92 (− 2.98, 1.14)	0.38

## Discussion

Reducing cockroach exposure in the home is recommended as an asthma management strategy [12] Adherence to this recommendation by caregivers of children with asthma is low and the reason for low adherence is unclear. The current study was conducted to address this data gap. We found that greater knowledge about cockroaches was associated with lower age at asthma diagnosis indicating that caregivers had less knowledge about the importance of cockroach exposure the older the child was when diagnosed. This is an important finding suggesting the need for greater emphasis on cockroach control in the home of older children with asthma. Greater concern about cockroach exposure (attitudes) was significantly correlated with the BMQ necessity scale. These results indicate that caregivers who are more concerned about the effect of cockroach exposure on their child’s asthma also have positive beliefs about the need for medication adherence. Therefore, caregivers with positive medication beliefs may be more receptive to cockroach interventions to control their child’s asthma. Cockroach practice score (reflecting taking action to promote remediation) was associated with both the attack management and attack prevention sub-scales of the PAMSE-13. These are promising results. Caregivers who indicated they had greater self-efficacy to manage and prevent asthma attacks reported practicing the actions required to reduce cockroach exposure. Taken together, the results indicate that the number of cockroaches in the home is most closely associated with how concerned (attitudes) caregivers are about cockroaches and their control as opposed to what they know (knowledge) or what they reported doing (practices) to prevent cockroaches.

These results reveal that higher practice scores did not significantly reduce cockroach exposure although participants in the higher quartiles had fewer cockroaches compared to those in the lowest quartile. This could be due to social desirability bias. Participants may have overestimated their responses on the practice scale due to perceived stigma around having pests in the home. Any bias would likely be non-differential since cockroach KAP was assessed prior to cockroach trapping and almost all participants reported seeing roaches in the home. Another reason may be that attitudes are a better indicator of health behavior than reported practices. This finding would align with previous research evaluating the HBM where perceived barriers and benefits (i.e. attitudes) are the strongest predictor of behavior change.

Another interesting finding is that the cockroach KAP subscales were not significantly correlated with asthma knowledge. This contrasts with the BMQ subscales (concern, overuse, and harm) which were moderately correlated with asthma knowledge, suggesting that improving asthma knowledge affects beliefs concerning asthma medications. However, the same is not true for cockroach control which is often overlooked as a strategy for managing asthma. Improving knowledge (either asthma or cockroach) may not be sufficient to increase concern (attitudes) or improve cockroach remediation practices. Finally, although participants in the highest quartile of total KAP had fewer cockroaches compared to those in the lowest quartile, the overall KAP score was not associated with reduced cockroach exposure. This suggests that the individual components may be more important than their sum.

Overall, the results provide information on key factors as targets for improving asthma management among caregivers with the intention of improving cockroach avoidance strategies. There appears to be patterns of different groups of caregivers which may require different avenues of support.

The study had several strengths. Participants were recruited from both community events and pulmonary clinics which allowed enrollment of participants specifically seeking care as well as those not seeking care. The cohort study was a prospective design and cockroach exposure was assessed after the assessment of cockroach KAP reducing the likelihood of information bias. In addition, actual cockroach exposure was assessed using cockroach counts. Limitations include lack of data on all sociodemographic factors that may influence both the home environment and cockroach exposure including household income, the small sample size which resulted in the inability to control for potential confounding factors, especially when assessing cockroach exposure, therefore the findings may be the result of residual confounding and should be considered with caution. Small sample size also resulted in reduced power. Finally, the use of convenience sampling may result in reduced generalizability.

## Conclusion

Generally, caregivers of children had positive scores on the cockroach KAP survey indicating positive heath behavior knowledge, attitudes, and practices. The hypothesis that greater knowledge, attitude, and practice scores would be associated with reduced cockroach exposure in the home was not entirely supported. Greater cockroach knowledge and practice scores were not associated with reduced cockroach exposure. Cockroach attitude scores appear to be driving exposure to cockroach in homes of children with asthma.

## Data Availability

The datasets used and/or analyzed during the current study are available from the corresponding author on reasonable request.

## Abbreviations

U.S.: United States
HBM: Health Beliefs Model
KAP: Knowledge, Attitudes, and Practices
BMQ: Beliefs about Medicine Questionnaire
PSS: Perceived Stress Scale
PAMSE-13: Parental Asthma Management Self-Efficacy
MOS-SSS: Medical Outcome Study Social Support Scale
